# The Global Burden of Migraine: From Headache Days to Migraine-Affected Days—A Real-World Perspective on the Human and Societal Impact of Migraine

**DOI:** 10.3390/medicina62101991

**Published:** 2026-10-15

**Authors:** Alin Ciubotaru, Albert Vamanu, Gabriela Popescu, Raluca Olariu, Andra Oancea, Cristina-Dora Cozma, Roxana Covali, Laura-Elena Cucu, Alexandra Mastaleru, Andrei Ionut Cucu, Florina Antochi, Alexandru Patrascu, Adina Maria Roceanu, Lucia Corina Dima-Cozma

**Affiliations:** 1 Grigore T. Popa University of Medicine and Pharmacy, 700115 Iasi, Romaniaraluca.olariu@umfiasi.ro (R.O.);; 2 Basic and Clinical Neuroscience Department, Institute of Psychiatry, Psychology and Neuroscience, King’s College London, London SE5 8AF, UK; albert.vamanu@kcl.ac.uk; 3 Emerald Hospital Iasi, 700115 Iasi, Romania; andrei.cucu@usm.ro; 4 Neurology Department, 05hrhxs64 University Emergency Hospital Bucharest, 020021 Bucharest, Romaniaamr2012mar@gmail.com (A.M.R.); 5 Apollonia University, 700511 Iasi, Romania; 6 Headache Group of Romanian Society of Neurology, 020021 Bucharest, Romania

**Keywords:** migraine, global burden of disease, migraine-related disability, interictal burden, quality of life, work productivity, calcitonin gene-related peptide, galcanezumab, preventive treatment, patient-reported outcomes

## Abstract

Migraine research counts prevalence, migraine days, headache days, attack frequency, years lived with disability and disability-adjusted life years. People living with migraine experience something wider: pain, nausea, sensory intolerance, cognitive slowing, cancelled arrangements, lost working time, disrupted family life and the anticipation of the next attack. This review examines the distance between what is measured and what is lived and asks how many days of life migraine actually affects and how many can be recovered. We summarise the epidemiological foundation provided by the Global Burden of Disease Study 2021, then set out a conceptual framework in which total migraine burden is the sum of attack burden, recovery burden and functional burden. We describe the attack as a multi-phase event running from premonitory symptoms through headache and associated features into postdrome and functional recovery and review the interictal burden of anticipation, avoidance and unpredictability, which affects days on which no pain occurs. Occupational, economic, family and social consequences are considered alongside the treatment gap that keeps burden high despite therapeutic progress. Galcanezumab is used as an illustrative treatment example to examine how reductions in migraine frequency may relate to broader functional and patient-reported outcomes in clinical trials and routine practice. Interictal burden correlates only negligibly to weakly with monthly migraine headache days, which suggests that interictal burden represents a dimension that is not fully captured by migraine-headache-day frequency and highlights the importance of measuring, reporting and interpreting complementary patient-reported and functional outcomes alongside frequency. The proposed “migraine-affected days” construct is presented as a conceptual and hypothesis-generating framework, rather than as a validated clinical or psychometric measure, diagnostic criterion, or established treatment endpoint.

## 1. Introduction

Migraine is among the most common neurological disorders and one of the leading causes of disability worldwide. In 2021, an estimated 1.2 billion people were living with migraine; headache disorders ranked as the second most prevalent condition globally and the third leading cause of years lived with disability (YLD), behind low back pain, major depressive disorder and age-related hearing loss [1]. These figures establish the scale of the problem. They describe how many people are affected and how much health loss is attributed to the condition at the population level.

What they describe less well is what a person with migraine actually experiences.

Migraine research counts prevalence, incidence, monthly migraine days (MMD), monthly headache days (MHD), attack frequency, attack duration, disability scores, YLD and disability-adjusted life years (DALY) [1]. A person with migraine experiences pain, nausea and vomiting, photophobia and phonophobia, cognitive slowing, an inability to work or drive, cancelled arrangements, disrupted family life, lost leisure time, fear of the next attack, unpredictability, a recovery period that continues after the pain resolves, and in many cases years of organising life around the disease.

The distance between these two lists is the subject of this review. Population-level estimates describe the magnitude of migraine. They do not fully describe what it means to live with migraine, and outcome measures inherited from population epidemiology do not automatically translate into the language patients use to judge whether treatment has worked.

The review proceeds in three movements. The first (Section 3.1, Section 3.2, Section 3.3, Section 3.4 and Section 3.5) establishes the epidemiological foundation and then widens the frame from headache days to affected days, tracing the attack across its phases, the interictal state, and the personal, family, occupational and economic consequences that follow. The second (Section 3.6, Section 3.7 and Section 3.8) examines why burden remains high despite therapeutic progress and what current outcome instruments do and do not capture. The third (Section 3.9 and Section 3.10) uses galcanezumab as an illustrative case to examine how reductions in migraine frequency may relate to functional, patient-reported and real-world outcomes. Galcanezumab is included as a worked example rather than as the subject of a comparative efficacy review.

Rationale for the study

The rationale for this review arises from a persistent discrepancy between how migraine is commonly measured and how the disease is experienced by patients.

Current clinical and epidemiological approaches provide robust and reproducible measures of disease frequency, particularly monthly migraine days and monthly headache days [2,3]. However, the burden of migraine extends beyond the days classified as migraine or headache days. An attack may involve premonitory symptoms before the onset of pain and postdromal impairment after the headache has resolved [3,4,5]. In addition, patients may experience anticipatory behaviour, avoidance of activities, uncertainty and functional restrictions during headache-free periods [6,7].

Consequently, the number of recorded migraine or headache days may not fully represent the number of days during which migraine affects an individual’s functioning and participation in everyday life.

This distinction is clinically relevant because the available evidence indicates that different dimensions of migraine burden are not interchangeable. Disability, quality of life, work productivity and interictal burden provide information that is not necessarily contained within a measure of headache frequency. In particular, the available evidence indicates only negligible-to-weak correlation between interictal burden and monthly migraine headache days, supporting the interpretation that these represent related but distinct dimensions of migraine burden.

These outcomes are often reported separately across different studies and are therefore difficult to interpret as components of a single patient-centred treatment benefit.

Elements of Novelty

The principal novelty of this review lies in its integrative approach to migraine burden. Rather than considering migraine frequency, disability, interictal burden, quality of life, productivity and treatment response as independent outcomes, the review examines how these domains are interconnected and how they collectively contribute to the real-world burden of migraine.

A further novel element is the proposal of “migraine-affected days” as a hypothesis-generating conceptual construct. The proposed framework extends the conventional counting of migraine or headache days by considering the broader period during which migraine may affect an individual’s functioning. It integrates three conceptually distinct components of burden: attack burden, reflecting pain and associated symptoms; recovery burden, reflecting premonitory and postdromal impairment; and functional burden, reflecting limitations and behavioural adaptations that may persist beyond periods of active headache.

A PubMed search conducted during preparation of this review did not identify prior use of the specific term “migraine-affected days” in the sense proposed here. The term is therefore introduced as a candidate construct for integrating dimensions of migraine burden that are currently considered across separate outcome domains. A candidate operationalisation is presented in Section 4.5 solely as a testable proposal and not as an established measurement definition.

The proposed construct is intended to provide a common interpretative framework through which existing evidence can be considered from the perspective of days of life affected and potentially recovered. It is not proposed as a replacement for established clinical endpoints and is not presented as a validated measurement instrument. Rather, it represents a conceptual and hypothesis-generating framework that may help guide future research toward outcomes that more closely reflect the patient’s experience of treatment benefit. The manuscript explicitly recognises that the proposed construct has not yet been operationalised or validated.

Finally, the review combines evidence from population epidemiology, clinical trials, patient-reported outcomes and prospective real-world cohorts. The use of galcanezumab as a worked example allows the relationship between reduction in migraine frequency and broader clinical and functional outcomes to be examined across different levels of evidence, without presenting galcanezumab as a comparative efficacy standard or implying superiority over other preventive treatments.

Aim of the study

The aim of this narrative review is to synthesise evidence on the multidimensional burden of migraine and to propose a conceptual framework of “migraine-affected days” that extends conventional migraine- and headache-day measures to include attack, recovery and functional burden.

Objectives

The objectives of this review are:

1. To characterise the multidimensional burden of migraine by examining attack-related symptoms together with premonitory and postdromal impairment, interictal burden, disability, occupational and household productivity, and social and family consequences.
2. To examine the limitations of conventional measures of migraine burden, particularly the use of monthly migraine days and monthly headache days as the principal indicators of treatment response, and to determine which clinically relevant dimensions of migraine remain insufficiently captured by these measures.
3. To evaluate the relationship between reduction in migraine frequency and broader patient-relevant outcomes, including disability, quality of life, work productivity, acute medication use, interictal burden and transition between episodic and chronic migraine.
4. To examine evidence from both controlled clinical trials and real-world studies in order to determine how the efficacy of preventive treatment translates into effectiveness and meaningful functional benefit in routine clinical practice.
5. To develop an integrative conceptual framework for understanding migraine-affected days, linking attack burden, recovery burden and functional burden, while considering the potential distinction between days on which headache is present and days on which migraine meaningfully affects the patient’s life.
6. To situate this framework against the endpoints currently recommended by the International Headache Society for controlled trials of preventive treatment, and to identify which domains are already covered and which are not.

## 2. Materials and Methods

### 2.1. Review Design

This article is a structured narrative review addressing the multidimensional burden of migraine and the extent to which conventional frequency-based outcomes capture the broader clinical and functional consequences of the disease.

A narrative review design was selected because the objective was to integrate evidence from different methodological settings and across multiple dimensions of migraine burden, rather than to estimate a single pooled treatment effect. The evidence considered includes epidemiological studies, population-based and clinic-based observational studies, randomised controlled trials, secondary analyses of clinical trials, health-economic studies and prospective real-world cohorts.

The review was structured around the distinction between migraine frequency and the broader period during which migraine may affect patients’ functioning, behaviour and participation in everyday life. Accordingly, evidence was synthesised across attack-related burden, recovery-related burden, functional impairment, interictal burden, quality of life, productivity and treatment-related outcomes.

Given the substantial heterogeneity in study populations, study designs, outcome definitions, follow-up periods and healthcare settings, a qualitative structured narrative synthesis was performed. No formal quantitative meta-analysis or pooled effect estimate was undertaken.

### 2.2. Review Scope and Conceptual Framework

To address the central research question set out in the Introduction, the available evidence was organised into three complementary domains of migraine burden:

Attack burden, including headache, associated symptoms, attack severity and duration;

1. Recovery burden, including premonitory and postdromal symptoms and impairment extending beyond the period of active headache; and
2. Functional burden, including disability, limitations in daily activities, occupational and household impairment, productivity loss, social and family consequences, avoidance and interictal burden.

This framework was used to organise and interpret the available evidence. The proposed “migraine-affected days” construct was considered as a conceptual framework intended to complement conventional migraine-day and headache-day measures. It was not treated as a validated clinical instrument, psychometric measure, diagnostic criterion or established treatment endpoint.

### 2.3. Information Sources

The literature search was conducted using three electronic bibliographic databases:

• PubMed/MEDLINE;
• Embase; and
• Scopus.

The electronic search was complemented by manual screening of the reference lists of relevant review articles and primary studies identified during the search process.

For the epidemiological component, evidence from the Global Burden of Disease (GBD) programme was also considered. The review used the most recent published GBD analysis of headache disorders available to the authors to provide the population-level epidemiological context for migraine burden.

The selection of these information sources was intended to capture evidence from clinical, epidemiological, functional, patient-reported and health-economic research relevant to the objectives of the review.

### 2.4. Search Strategy

Reference lists of relevant publications were subsequently screened manually to identify additional studies contributing to the predefined domains of the review.

For PubMed/MEDLINE, the search strategy combined Medical Subject Headings (MeSH) and free-text terms addressing migraine and its multidimensional clinical, functional and societal burden. The Boolean operators AND and OR were used to combine the search concepts. The PubMed/MEDLINE search strategy was:

*((“Migraine Disorders”[Mesh] OR migraine[tiab] OR “chronic migraine”[tiab]) AND (“Disease Burden”[Mesh] OR burden*[tiab] OR disability[tiab] OR “Quality of Life”[Mesh] OR “quality of life”[tiab] OR interictal[tiab] OR premonitory[tiab] OR postdrom*[tiab] OR “work productivity”[tiab] OR productivity[tiab] OR absenteeism[tiab] OR presenteeism[tiab] OR “medication overuse”[tiab] OR “patient-reported outcome*”[tiab] OR “attack duration”[tiab] OR “migraine days”[tiab] OR “treatment persistence”[tiab] OR “real-world”[tiab] OR galcanezumab[tiab] OR CGRP[tiab]))**.

The search was conducted from database inception to 1 September 2026. No date restrictions were applied. No language restriction was imposed at the electronic search stage; however, only English-language reports were ultimately included in the narrative synthesis.

The last search was run on 1 September 2026. No language restriction was applied at the search stage, although only English-language reports were ultimately included. Searches were not date-limited, so that foundational instrument-validation and epidemiological sources published before 2000 remained eligible. As this was a structured narrative review rather than a systematic review, no duplicate-independent screening procedure or PRISMA-based quantitative accounting of records was undertaken. The implications of this approach are described in Section 2.6 and Section 2.11.

### 2.5. Eligibility Criteria

The review considered peer-reviewed epidemiological studies, population-based and clinic-based cohort studies, randomised controlled trials, prespecified secondary analyses and post hoc/exploratory analyses of clinical trials, health-economic analyses and prospective real-world cohorts.

Studies were considered relevant when they provided evidence concerning one or more of the following domains:

• Migraine or headache frequency;
• Attack characteristics and associated symptoms;
• Premonitory or postdromal burden;
• Disability and functional impairment;
• Health-related quality of life;
• Occupational and household functioning;
• Absenteeism, presenteeism and work productivity;
• Interictal burden;
• Acute medication use and medication overuse;
• Treatment persistence;
• Transition between episodic and chronic migraine; or
• Clinical effectiveness of preventive treatment in routine practice.

Case reports were excluded. Conference abstracts without subsequent full publication and non-peer-reviewed commentaries were generally excluded. Where an abstract represented the only available report of an otherwise identifiable dataset directly relevant to the review, this was retained and its limited publication status was taken into account when interpreting the evidence.

No restriction to a single study design was imposed because the review was intended to integrate evidence from complementary methodological approaches.

### 2.6. Identification and Selection of Relevant Evidence

Potentially relevant publications were identified through the electronic database searches and manual examination of reference lists. Titles and abstracts were assessed for relevance to the predefined domains of migraine burden, followed by consideration of the full text of publications judged potentially relevant.

Study selection was performed by the review authors on the basis of relevance to the predefined scope and conceptual framework of the review. No formal duplicate-independent screening procedure was used. Where relevance or eligibility was uncertain, no formal adjudication algorithm was applied; such decisions were made on the basis of the predefined domains and the authors’ judgement regarding the contribution of the publication to the review framework.

Because this was a structured narrative review rather than a systematic review, no PRISMA flow diagram was constructed, and no formal quantitative accounting of records identified, screened, excluded or included was undertaken.

### 2.7. Data Extraction and Organisation of Evidence

The extracted evidence was subsequently organised according to the three complementary domains defined in Section 2.2:

Attack burden: headache, associated symptoms, attack severity and duration.

Recovery burden: premonitory and postdromal symptoms and functional impairment extending beyond the period of active headache.

Functional burden: disability, limitations in daily activities, occupational and household impairment, productivity loss, social and family consequences, avoidance and interictal burden.

Treatment-related outcomes were extracted across these domains and included changes in migraine frequency, disability, acute medication use, treatment persistence, transition between episodic and chronic migraine, and real-world treatment effectiveness.

### 2.8. Narrative Synthesis

A structured qualitative narrative synthesis was performed.

Epidemiological studies were used to characterise the population-level magnitude and distribution of migraine. Clinical and observational studies were considered for evidence concerning attack burden, disability, functional impairment and interictal burden. Randomised controlled trials were used primarily to characterise treatment efficacy under controlled conditions, whereas prospective real-world studies were considered in relation to treatment effectiveness, persistence, tolerability and outcomes observed in routine clinical practice. These evidence streams were interpreted separately because observational studies do not provide the same basis for causal inference as randomised controlled trials.

### 2.9. Interpretation of Different Types of Evidence

The evidence was interpreted according to study design and analytical status rather than treated as a single evidentiary body. For treatment-related conclusions, prespecified primary outcomes from randomised controlled trials were considered the principal evidence for efficacy. Prespecified secondary outcomes from randomised controlled trials were interpreted as complementary evidence addressing additional clinically relevant domains, including disability, quality of life and functional outcomes. Post hoc, exploratory or other analyses conducted outside the prespecified primary or secondary outcome structure were interpreted as hypothesis-generating and were not considered equivalent to prespecified trial outcomes. Prospective observational real-world studies were used to assess treatment effectiveness, persistence and outcomes observed in routine clinical practice but were interpreted separately from randomised evidence because their observational design does not provide the same basis for causal inference.

The analytical status of individual findings was based on the classification reported in the original publication. The review did not reclassify an analysis as prespecified, secondary or post hoc when this status was not clearly reported by the source.

This hierarchy represents an interpretative framework for the narrative synthesis rather than a formal GRADE assessment or quantitative ranking of the evidence. Accordingly, evidence from different designs was not assigned a numerical quality score.

Because this was a structured narrative review, no formal risk-of-bias instrument was applied across the included evidence. Consequently, the review does not provide a pooled estimate of the treatment effect or a formal quantitative assessment of certainty of evidence.

### 2.10. Galcanezumab as an Illustrative Case

Galcanezumab was selected as an illustrative therapeutic example through which the relationship between reduction in migraine frequency and broader patient-relevant outcomes could be examined.

The selection was based on the availability of evidence across several clinically relevant populations and outcome domains, including episodic migraine, chronic migraine, patients with previous failure of multiple preventive treatment categories, functional outcomes, interictal burden and prospective real-world effectiveness.

Galcanezumab was therefore used as a worked example within the broader framework of migraine burden rather than as the subject of a comparative systematic review of preventive therapies. Its inclusion was intended to illustrate how reduction in migraine frequency may relate to changes in disability, function, interictal experience and other patient-relevant outcomes and does not imply superiority over other CGRP-targeted or conventional preventive treatments.

### 2.11. Methodological Limitations of the Review Approach

This review was conducted as a structured narrative review rather than as a systematic review or meta-analysis. Accordingly, no review protocol was prospectively registered, study selection was not performed using duplicate-independent screening, no PRISMA flow diagram was constructed, and no formal quantitative accounting of records identified, screened, excluded or included was undertaken. A formal risk-of-bias instrument was also not applied across the heterogeneous body of evidence.

The selection of evidence therefore involved author judgement regarding the relevance of individual publications to the predefined domains and conceptual framework of the review. Although the search strategy included multiple bibliographic databases and manual screening of reference lists, the absence of duplicate-independent screening and quantitative accounting means that the possibility of selection bias or omission of potentially relevant literature cannot be excluded.

The heterogeneity of the evidence base represents an additional methodological consideration. The included studies differed in population characteristics, study design, outcome definitions, follow-up periods and healthcare settings, precluding a quantitative synthesis or direct comparison across all evidence domains.

The proposed concept of “migraine-affected days” should similarly be interpreted as a conceptual and hypothesis-generating framework emerging from the synthesis of existing evidence. It is not presented as a validated measurement instrument and requires future operationalisation and empirical validation before it can be considered for use as a formal clinical or research endpoint.

## 3. Results

### 3.1. Global and Epidemiological Burden of Migraine

The Global Burden of Disease (GBD) 2021 analysis provides the most recent population-level estimates considered in this review and demonstrates the substantial global burden associated with migraine and other headache disorders. In 2021, approximately 1.2 billion people were estimated to be living with migraine worldwide. Headache disorders collectively ranked as the second most prevalent condition globally and represented the third leading cause of years lived with disability (YLD), highlighting the substantial contribution of headache disorders to global non-fatal health loss [1].

The distribution of migraine-related burden was not uniform across age and sex groups. The highest burden was observed among women aged 30–44 years [1]. This concentration of burden in working-age adults is particularly relevant because migraine affects individuals during periods commonly characterised by substantial occupational, family and social responsibilities.

Temporal analyses from the GBD 2021 study showed that absolute numbers of cases and YLD increased between 1990 and 2021, whereas age-standardised rates remained broadly stable [1]. This pattern suggests that the increase in the absolute population burden was predominantly related to demographic changes, including population growth and ageing, rather than to a substantial increase in age-standardised individual risk [1].

The GBD 2021 analysis establishes migraine as a major contributor to global neurological morbidity and disability, with substantial variation across age, sex and healthcare-access settings [1]. Absolute burden increased over the period 1990–2021, whereas age-standardised rates remained comparatively stable, indicating an important contribution of demographic change to the increase in the number of affected individuals [1]. The principal quantitative estimates are summarised in Table 1.

Overall, the epidemiological evidence establishes migraine as a highly prevalent neurological disorder and a major contributor to population-level disability. However, these population-level measures primarily quantify prevalence and health loss and do not directly capture the distribution of individual days affected by migraine, the temporal extension of an attack beyond the headache phase, or the functional consequences occurring between attacks (Table 1).

### 3.2. Migraine Days as a Conventional Measure of Disease Burden

Monthly migraine days (MMD) and monthly headache days (MHD) are widely used frequency-based measures for characterising migraine burden and evaluating the response to preventive treatment. Their use provides a reproducible and clinically interpretable estimate of the frequency of migraine or headache occurrence and allows changes in disease frequency to be compared across populations and treatment periods.

The burden experienced during an individual migraine day may also vary substantially according to attack duration and pain intensity. In a post hoc analysis of the phase 3 galcanezumab clinical development programme, total pain burden was assessed using a severity-weighted duration measure that incorporated the duration and maximum pain intensity of migraine headache. After accounting for migraine headache-day frequency and maximum pain severity, migraine duration on individual days accounted for the largest unique proportion of variability in total pain burden, representing 57.4% in episodic migraine and 61.1% in chronic migraine [2]. Thus, an identical number of migraine headache days may be associated with different overall pain burdens depending on the duration of individual attacks.

Taken together, the evidence demonstrates that MMD and MHD provide an important measure of migraine frequency but do not encompass the full temporal and functional burden of the disease. The available evidence identifies several additional dimensions including attack duration and intensity, premonitory and postdromal symptoms, interictal anxiety and avoidance that may vary independently from the number of recorded migraine days. Consequently, frequency-based measures should be interpreted as one component of migraine burden rather than as a comprehensive measure of the patient’s overall experience of the disease (Table 2).

### 3.3. Attack, Premonitory and Postdromal Burden

The clinical burden of a migraine attack is not necessarily restricted to the period of active headache. The available evidence describes migraine as a temporally evolving process that may include symptoms before headache onset and a recovery phase after resolution of headache. These phases provide additional information regarding the duration and clinical impact of an individual migraine episode beyond the conventional count of migraine or headache days [3,4].

The premonitory phase may precede the onset of headache by hours and may include a range of non-pain symptoms. Reported manifestations include fatigue, changes in mood or activity, difficulty concentrating, neck stiffness, food cravings, repetitive yawning and increased sensitivity to light or sound [3,4]. These symptoms may occur before the onset of the headache phase and therefore are not necessarily represented by measures that define a migraine day according to the presence of headache. Quantitative studies consistently demonstrate that premonitory symptoms are common, while also showing substantial variation according to population, ascertainment method and symptom definition [9,10]. Across the available evidence, fatigue, neck symptoms, concentration difficulties and sensory symptoms are among the most frequently reported manifestations [4,9,10,11]. The quantitative estimates from these studies are summarised in Table 3.

The International Classification of Headache Disorders, 3rd edition (ICHD-3), recognises premonitory symptoms as part of the clinical evolution of migraine attacks and describes a postdromal phase following resolution of headache [3].

These estimates should not be interpreted as a single population-wide prevalence figure. Differences in study setting, participant selection, symptom definitions and methods of enquiry substantially influence the proportion of individuals reporting premonitory symptoms. In particular, clinic-based and event-based studies may identify higher frequencies than population-based studies [9,10].

The postdrome may last for up to 48 h and commonly includes fatigue, changes in mood and cognitive difficulties [3]. Thus, the temporal boundaries of a migraine episode may extend beyond the period during which the patient experiences the principal headache symptoms.

Prospective diary-based and observational evidence indicates that postdromal symptoms are common and may persist after headache resolution, with fatigue, cognitive difficulties, mood changes and neck symptoms among the most frequently described manifestations [5,12]. The available estimates vary substantially across study designs and populations, reflecting differences in definitions, ascertainment methods and risk of bias [12]. Key quantitative findings are summarised in Table 3.

Functional recovery did not occur immediately after headache resolution in all attacks. In the same prospective study, return to normal was achieved within 24 h after spontaneous resolution of headache in 93% of attacks [5]. Importantly, overall health-state scores remained lower during the postdrome than during the interictal period, indicating that symptom resolution and return to the patient’s usual level of functioning were not necessarily simultaneous [5].

The study also found no relationship between the medication taken for the acute headache and the duration of the postdrome, and migraine severity was not associated with postdrome duration [5]. These findings are relevant because they suggest that the persistence of postdromal symptoms cannot simply be attributed to differences in acute treatment or to the intensity of the preceding headache.

The premonitory and postdromal findings summarised above are set out in Table 3.

### 3.4. Interictal Burden

The burden of migraine may persist during periods without active headache. Interictal burden refers to health or well-being losses attributable to migraine that are experienced while the patient is free of headache and includes symptoms, anxiety, avoidance behaviour, functional limitations and cumulative consequences occurring between attacks [6].

The Eurolight findings are important population-based European evidence, but their estimates should not be interpreted as globally representative. More recent studies from non-European populations provide complementary evidence that interictal burden is also clinically relevant outside Europe, although differences in sampling and measurement instruments limit direct quantitative comparison across settings.

Evidence from large non-European populations provides complementary information. In the population-based OVERCOME (Japan) study, which included 17,071 respondents with migraine, 41.5% experienced moderate-to-severe interictal burden according to the MIBS-4. Among individual MIBS-4 domains, 26.8% of respondents reported that they worried about planning social or leisure activities at least some of the time between headache days, while 19.0% reported that headaches affected work or school at times when they did not have a headache [13]. A subsequent second-wave OVERCOME study conducted in Japan in 2023 included 19,590 adults with migraine and found that 47.1% had MIBS-4 scores ≥3, indicating moderate-to-severe interictal burden. Higher interictal burden was associated with greater disability, poorer migraine-specific quality of life and greater work and activity impairment, including among participants with lower ictal burden [14].

The broader literature therefore suggests that interictal burden is not restricted to the European setting. A systematic review published in 2026 identified 29 studies evaluating instruments or measurement properties relevant to migraine interictal burden. The MIBS-4 was used in 26 studies, compared with one study using the Eurolight questionnaire and two using the Fear of Attacks Inventory. The evidence base included large cohorts from Japan, the United States, the European Union and Spain, although the authors noted that evidence for several psychometric properties remains limited [15].

Taken together, these findings support interictal burden as a clinically relevant dimension of migraine, while also indicating that estimates obtained with different instruments and in different populations should not be assumed to be directly interchangeable.

Patient-reported assessment provides further evidence that interictal burden represents a dimension that is related to, but not synonymous with, migraine frequency. In a phase 3 preventive-treatment population of 462 participants, the Migraine Interictal Burden Scale (MIBS-4) was associated with migraine-specific quality of life, depressive symptoms and headache-related disability. In contrast, its correlation with monthly migraine headache days was low, with Spearman correlation coefficients of 0.21 at baseline and 0.32 at month 6 [7]. This pattern indicates that the number of migraine headache days alone does not fully account for the burden experienced between attacks.

The MIBS-4 evaluates several clinically relevant aspects of the interictal experience, including impairment in work or school, interference with family and social life, difficulties making plans or commitments, and emotional or cognitive consequences. Its use therefore provides a patient-centred approach to assessing consequences that may remain clinically relevant despite the absence of active headache.

The interictal findings discussed in this section are summarised in Table 4.

### 3.5. Functional, Occupational and Social Burden

The burden of migraine extends beyond the occurrence of headache and includes measurable impairment in daily activities, occupational functioning, household responsibilities and social participation. Patient-reported outcome measures used in migraine research capture several of these dimensions, including disability, headache impact, health-related quality of life and productivity. Among the instruments most frequently used for this purpose are the Migraine Disability Assessment (MIDAS), the Headache Impact Test-6 (HIT-6) and migraine-specific quality-of-life measures [16,17,18].

The MIDAS instrument quantifies disability according to the number of days during which migraine or headache interfered with work or school, household activities and leisure activities over a three-month recall period. The instrument therefore captures consequences of migraine in several functional roles rather than focusing exclusively on headache frequency [16]. HIT-6 provides a complementary assessment of headache impact, including effects on pain, daily activities, energy, emotional functioning and ability to concentrate [17]. Migraine-specific quality-of-life instruments further assess the effect of migraine on role functioning and emotional well-being [18].

Observational evidence demonstrates substantially greater functional impairment among patients with chronic migraine than among those with episodic migraine, across missed work or school, reduced productivity, disability, headache impact and general health-related quality of life [19]. The quantitative estimates are presented in Table 5.

The same study demonstrated an impact on broader health-related quality of life. Mean general health scores on the SF-36 were 39.9 ± 14.7 among participants with chronic migraine and 66.2 ± 18.2 among those with episodic migraine (*p* = 0.001) [19]. These findings illustrate that increasing migraine frequency is associated with a broader deterioration in functional status and perceived health, although frequency alone does not specify the mechanisms through which this impairment occurs.

The occupational consequences of migraine are also reflected at the population level. The economic consequences of migraine are also substantial, with indirect productivity losses representing an important component of the total societal burden [20]. The principal quantitative estimates are summarised in Table 5.

Evidence from the Eurolight project further demonstrated that lost productive time is associated with the broader interictal burden of headache. Participants with migraine reported consequences extending beyond the immediate period of headache, including reluctance to disclose their condition to others, difficulties with educational performance, reduced earnings and career consequences [6]. Thus, the functional burden of migraine includes both direct impairment during headache episodes and cumulative consequences that may develop across repeated attacks and the periods between them.

The relationship between headache frequency and lost productivity has also been examined using individual participant data from population-based studies. Analyses incorporating headache frequency, usual attack duration, headache intensity and productivity loss indicate that these clinical characteristics jointly contribute to the amount of paid and household work lost because of headache [21]. This supports the interpretation that frequency is an important determinant of productivity loss but does not operate independently of other characteristics of the headache experience.

The consequences also extend into family life, career trajectory and financial security. In the CaMEO study, among 13,064 respondents, 32.7% reported that headache had negatively affected at least one career area, rising from 30.3% in episodic migraine to 58.4% in chronic migraine, and 32.1% endorsed worry about long-term financial security because of migraine, rising from 29.7% to 57.4% across the same comparison. Among respondents in a cohabiting relationship, 3.2% reported that they had chosen not to have children, had delayed having children or had had fewer children because of migraine, rising from 2.6% in episodic migraine to 9.5% in chronic migraine [22]. These outcomes represent decisions not taken rather than activities interrupted, and no headache diary records them.

The economic consequences extend to unpaid as well as paid work. A German population-based analysis monetised productivity losses as gross value added and identified losses of tens of billions of euros annually in paid work, with a comparable magnitude in unpaid work such as household and caring activity [23]. Because unpaid work is disproportionately performed by the demographic group in which migraine burden peaks, its omission from cost models introduces a distributional error as well as an underestimate.

Stigma has recently been operationalised in this field and can therefore be treated as evidence rather than impression. The Migraine-Related Stigma questionnaire, developed in a population-based sample of 61,932 United States adults with migraine, resolves into two internally consistent subscales: an external perception of secondary gain and an external perception that others minimise the burden of the disease [24]. The second follows directly from a disease label that non-sufferers map onto their own experience of ordinary headache, and it plausibly contributes to the disclosure reluctance recorded in the Eurolight data.

Taken together, the reviewed evidence demonstrates that migraine affects several domains of functioning, including employment, productivity, household activities, education, social participation and perceived health. The magnitude of these consequences increases substantially with greater disease burden, particularly in chronic migraine, but the available evidence also indicates that functional impairment cannot be reduced to a simple count of headache days. Frequency, attack characteristics, interictal consequences and individual functional circumstances contribute to the overall impact experienced by patients (Table 5).

The quantitative estimates presented in Table 5 are intended to illustrate the range of functional and health-related consequences reported in the literature rather than to provide globally representative prevalence estimates. In particular, several of the clinical disability and productivity estimates derive from a tertiary headache-centre population and should therefore be interpreted in the context of the study setting and population characteristics.

### 3.6. Treatment Gaps, Disease Progression and Medication Overuse

The population-level burden described in Section 3.1 persists despite the availability of effective preventive treatment. Two processes account for much of this: a large proportion of eligible patients never receive prevention, and a proportion of those who remain untreated progress to higher attack frequencies.

Consultation is the first filter. In the CaMEO study, among participants meeting criteria for chronic migraine with headache-related disability, 512 of 1254 (40.8%) reported currently consulting a healthcare professional for headache, and accurate diagnosis and appropriate treatment act as successive further filters on the eligible population [25].

The scale of the preventive gap has been quantified across several countries. Population data have long indicated that a substantial minority of people with migraine meet criteria for preventive treatment while only a small fraction receive it [26]. The CaMEO-International study surveyed 90,613 people across six countries, of whom 14,492 met criteria for migraine and completed the full survey. Applying the American Headache Society 2021 consensus algorithm, 36.2% qualified for preventive treatment, and of those, 84.5% were not taking a preventive medication at the time of the survey [27].

A second gap is easily overlooked. Among respondents in that study who were currently taking a preventive medication, 50.2% still met the algorithm criteria for needing preventive treatment [27], indicating inadequate benefit from the regimen they were already receiving. The treatment gap is therefore a gap in effectiveness among the treated as well as a gap in prescribing.

The OVERCOME study reached a compatible conclusion in a United States sample of 59,001 participants, among whom 77.1% were candidates for initiation or modification of prescription treatment. Acute medication overuse was most frequent in the group with combined acute and preventive needs, at 37.0%, and moderate-to-severe interictal burden was reported by 69.9% of that group [28]. Severe interictal burden was most strongly associated with unmet acute treatment need, whereas acute medication overuse was most strongly associated with unmet preventive need [28].

Persistence on conventional oral preventives contributes to the gap. In a retrospective analysis of 8707 patients with chronic migraine, persistence with the initial oral preventive medication was 25% at six months and 14% at twelve months, and persistence declined further among patients who switched or re-initiated treatment [29]. A majority of patients therefore cycle through preventive medications without remaining on any of them.

Attack frequency is not static. Longitudinal population data indicate that a small proportion of people with episodic migraine transition to chronic migraine each year, with baseline headache frequency among the strongest predictors and with risk rising as baseline frequency increases [30]. Frequent acute medication use is both a rational response to frequent attacks and an established risk factor for progression [30]. Frequent acute medication use is both a rational response to frequent attacks and an established epidemiological risk factor for migraine progression, which may contribute to a cycle in which frequent reliance on acute medication is associated with an increased risk of migraine progression.

Reductions in acute medication days and cessation of medication overuse therefore act on progression risk as well as on symptom control, which is relevant to the interpretation of the real-world findings reported in Section 3.10.

### 3.7. Limitations of Frequency-Based Clinical Endpoints

The reviewed evidence indicates that the principal instruments used to assess migraine burden capture distinct and clinically relevant dimensions of the disease. Monthly migraine days (MMD) and monthly headache days (MHD) quantify the frequency of attacks, whereas disability, quality of life, productivity and interictal burden require complementary patient-reported or functional measures. The available instruments therefore provide partially overlapping but non-equivalent information about the patient’s experience of migraine.

The Migraine Disability Assessment (MIDAS) quantifies days of lost or substantially reduced productivity in work or school, household activities and social or leisure activities over a three-month recall period [16]. The Migraine-Specific Quality of Life Questionnaire version 2.1 (MSQ v2.1) evaluates the effects of migraine on role-function restrictive, role-function preventive and emotional-function domains [18]. These instruments therefore extend assessment beyond headache frequency by addressing consequences of migraine for everyday functioning and quality of life.

The distinction between frequency and broader burden is particularly relevant when treatment response is defined primarily according to a reduction in MMD. A statistically significant reduction in migraine days demonstrates improvement in the frequency domain, but it does not by itself establish the extent to which disability, work productivity, acute medication reliance, interictal burden or social functioning have improved. These outcomes may change in parallel with migraine frequency, but they represent separate dimensions that require direct measurement.

Importantly, this should not be interpreted to mean that contemporary preventive-treatment trials assess migraine frequency in isolation. Current International Headache Society recommendations explicitly recognise responder rates, acute medication use, headache-related disability and health-related quality of life as complementary outcomes alongside the primary frequency endpoint [8,31]. The methodological gap identified here is therefore not the absence of multidimensional outcomes from contemporary trial guidance, but the extent to which these outcomes are consistently measured, reported, interpreted together and incorporated into an overall assessment of treatment benefit.

The available evidence provides empirical support for this distinction. As described above, interictal burden measured using the MIBS-4 showed only low correlations with monthly migraine headache days, with Spearman correlation coefficients of 0.21 at baseline and 0.32 at month 6 [7]. Thus, the number of migraine headache days cannot be considered a reliable surrogate for the burden experienced between attacks.

A similar limitation applies to response thresholds based exclusively on frequency. A patient achieving a predefined reduction in MMD may appropriately be classified as a treatment responder with respect to the frequency endpoint while continuing to experience substantial residual burden in other domains. For example, the patient may continue to experience severe individual attacks, require acute medication, experience anticipatory anxiety or retain functional limitations despite a clinically meaningful reduction in migraine days. These domains are not incorporated into the frequency endpoint and therefore cannot be inferred reliably from the responder classification alone.

The reviewed literature also indicates that several additional measures can provide information complementary to MMD and MHD. Acute medication days quantify the frequency with which patients require symptomatic treatment. Measures of attack severity and duration characterise the intensity and temporal extent of individual attacks. MIDAS and MSQ provide information on disability and health-related quality of life, while the MIBS-4 addresses interictal burden [7,16,18]. Work productivity instruments such as the Work Productivity and Activity Impairment questionnaire provide a further distinction between absenteeism and presenteeism.

From a measurement perspective, migraine-day frequency therefore remains an appropriate core outcome for preventive treatment studies because it is reproducible, clinically interpretable and directly responsive to treatment. However, a comprehensive assessment of treatment benefit requires additional outcomes capable of capturing disability, quality of life, productivity, acute medication use and interictal burden. The evidence reviewed in the preceding sections supports the need for such multidimensional assessment without implying that the conventional migraine-day endpoint should be replaced (Table 6).

### 3.8. Treatment-Related Reduction in Migraine Days and Patient-Reported Outcomes

The reviewed evidence indicates that preventive treatment can reduce the frequency of migraine days while also producing measurable changes in patient-reported disability, quality of life and functional burden. These outcomes are related but distinct, supporting the use of complementary endpoints when evaluating the clinical consequences of preventive therapy.

Overall, the available evidence supports MMD reduction as a valid core endpoint for preventive treatment while demonstrating the need for complementary patient-reported and functional outcomes. The magnitude of change in migraine-day frequency should therefore be interpreted together with changes in disability, quality of life, productivity and interictal burden when the objective is to characterise the broader clinical benefit of preventive treatment (Table 7).

### 3.9. Galcanezumab as an Illustrative Clinical Trial Example

Galcanezumab was selected as an illustrative example because its clinical development programme provides evidence across both conventional frequency-based outcomes and several patient-reported and functional domains. The phase 3 programme included trials in episodic migraine (EVOLVE-1 and EVOLVE-2), chronic migraine (REGAIN), and patients with previous failure of multiple preventive-treatment categories (CONQUER) [32,33,34,35].

Across these studies, galcanezumab reduced migraine or headache frequency compared with placebo and was associated with improvements in migraine-specific quality of life and disability. Pooled analyses further showed that greater reductions in migraine-headache-day frequency were accompanied by greater improvements in patient-reported outcomes [36,37]. Importantly, these findings do not imply that functional benefit can be inferred from frequency reduction alone; disability and quality-of-life outcomes remained independently measurable domains [36,37].

Galcanezumab is therefore used in this review only to illustrate how reduction in migraine frequency can coexist with changes in disability and patient-reported functioning. It is not intended to serve as a comparator or as a representative estimate of the effects of preventive treatment as a whole.

### 3.10. Galcanezumab: Real-World Evidence as an Illustrative Example

Real-world evidence provides a complementary perspective because treatment effectiveness may differ from efficacy observed under controlled trial conditions. In the GARLIT cohort, treatment with galcanezumab was associated with reductions in migraine frequency together with improvements in pain intensity, acute medication use, headache impact and disability, and a substantial proportion of patients with chronic migraine transitioned to an episodic pattern during follow-up [38,39].

These findings are consistent with the broader observational literature on CGRP-targeted monoclonal antibodies. A systematic review and meta-analysis of 47 observational cohort studies reported clinically relevant reductions in monthly migraine and headache days, although the absence of placebo-controlled comparators limits causal interpretation of these estimates [40].

Within the present review, these real-world findings are used to illustrate how frequency reduction may be accompanied by changes in disability, medication use and functional burden in routine practice. They are not intended to establish the superiority of galcanezumab or to represent the effectiveness of all preventive therapies.

Randomised controlled trials and prospective observational cohorts address related but distinct questions. Randomised trials provide the principal evidence for treatment efficacy under controlled conditions, whereas prospective real-world studies provide complementary evidence regarding treatment effectiveness, persistence and clinical outcomes in routine practice. Because observational cohorts are susceptible to confounding, selection effects and differences in treatment access or background care, their findings were interpreted as complementary effectiveness evidence rather than as equivalent to randomised estimates of treatment efficacy.

## 4. Discussion

### 4.1. Principal Findings and Interpretation

The principal finding of this review is that migraine burden cannot be adequately characterised by counting migraine days alone. Across the evidence reviewed, the clinical consequences of migraine extend across several temporally and functionally distinct domains, including the symptomatic phases surrounding an attack, the interictal period, disability, occupational and household productivity, social participation and health-related quality of life. The number of days on which a patient fulfils criteria for a migraine day therefore represents an important component of disease burden, but not its complete expression.

This distinction is supported by evidence from different methodological approaches. Electronic-diary studies demonstrate that clinically relevant symptoms may occur before and after the headache phase [4,5]. Population-based data demonstrate that patients may experience symptoms, anxiety and avoidance during periods without headache [6]. Patient-reported outcome studies further show that interictal burden is only weakly correlated with monthly migraine headache days [7]. Together, these findings indicate that temporal exposure to migraine and its consequences is broader than the period captured by conventional headache-day definitions.

This finding requires a caveat that is easily missed. In the same analysis, MIBS-4 correlated moderately with depressive symptoms measured on the PHQ-9 and with migraine-specific quality of life, while correlating only weakly with attack frequency [7]. An obvious alternative interpretation is therefore that the interictal scale is capturing comorbid depression and anxiety rather than a migraine-specific burden, in which case the dissociation from migraine-day counts would be an artefact rather than a discovery.

The available data do not separate these interpretations. Migraine and depression are bidirectionally associated, so shared variance is expected on either account, and correlations between patient-reported measures completed by the same participants cannot establish which construct is primary. Future studies should therefore assess interictal burden together with validated measures of depression and anxiety and incorporate these variables as covariates in multivariable analyses. At minimum, this approach should be applied in populations not selected solely for previous preventive failure; where feasible, analyses should additionally account for other clinically relevant sources of functional impairment, including sleep disturbance, other chronic pain conditions and major medical or psychiatric comorbidities. A non-migraine comparison group could provide further information on the specificity of interictal burden, although such a design would not by itself establish migraine attribution. Until these analyses are available, the present findings should be interpreted conservatively: interictal burden is not adequately represented by monthly migraine headache days, but the proportion of this burden that is specifically attributable to migraine rather than affective or other comorbid factors remains uncertain. The chronic migraine trial guidelines already recommend assessment of comorbid depression and anxiety [31], supporting their inclusion as covariates in future validation studies.

The same issue applies directly to the proposed “migraine-affected days” construct. A headache-free day characterised by reduced activity, avoidance or anticipatory limitation should not automatically be attributed to migraine solely on the basis of patient-reported functional restriction. Construct validation will therefore require simultaneous assessment of affective and other comorbid conditions, allowing the migraine-attributable component of interictal impairment to be distinguished from impairment that persists independently of migraine. This distinction is particularly important for establishing discriminant validity and for determining whether the proposed construct provides information beyond established measures of depression, anxiety, disability, quality of life and monthly migraine-day frequency.

The treatment evidence should not, however, be interpreted as a single evidentiary block. The most direct evidence for galcanezumab efficacy comes from prespecified primary frequency outcomes in randomised controlled trials [32,33,34,35]. Prespecified secondary outcomes assessing disability, migraine-specific quality of life and related patient-reported domains provide complementary evidence that treatment effects extend beyond frequency alone [34,37]. Pooled or exploratory analyses examining relationships between frequency response and patient-reported outcomes provide additional supportive evidence, but these findings should be interpreted more cautiously than prespecified primary outcomes because they address associations between outcome domains rather than the primary efficacy question [36]. Where analyses were post hoc or exploratory, their findings are considered hypothesis-generating rather than confirmatory.

Prospective real-world studies provide a different type of evidence. The GARLIT cohorts demonstrate that reductions in migraine frequency may be accompanied in routine practice by changes in acute medication use, headache impact, disability and migraine chronicity [38,39]. These observations are clinically relevant for treatment effectiveness and generalisability, but their observational design prevents them from being interpreted as having the same causal evidentiary status as randomised controlled trials.

Accordingly, the different evidence streams are complementary rather than interchangeable: prespecified primary randomised outcomes provide the principal efficacy evidence; prespecified secondary outcomes extend the clinical characterisation of treatment benefit; post hoc or exploratory analyses generate hypotheses concerning relationships between outcomes; and real-world observational studies inform effectiveness and applicability in routine practice.

Controlled and real-world preventive-treatment studies, including the galcanezumab programme used here as an illustrative example, demonstrate that reductions in migraine frequency may be accompanied by improvements in disability, migraine-specific quality of life, acute medication use and interictal burden [32,33,34,35,36,37,38,39,40]. These domains nevertheless remain distinct and require independent assessment.

These findings support the clinical relevance of frequency reduction while simultaneously demonstrating that treatment benefit can be observed across several independent outcome domains.

Importantly, the evidence does not support treating these domains as interchangeable. A reduction in monthly migraine days is a direct measure of reduced attack frequency. A reduction in MIDAS reflects reduced disability; an improvement in MSQ reflects better migraine-specific functioning and quality of life; and a reduction in MIBS-4 reflects lower burden during the interictal period [7,16,18]. Each measure therefore captures a different component of the patient’s experience. The observation that these outcomes frequently improve together should not be interpreted as evidence that one can be calculated reliably from another.

This distinction also identifies an important research gap. The evidence is relatively strong for the individual components of migraine burden but substantially weaker for the direct measurement of what patients regain after successful preventive treatment. Clinical trials routinely quantify days reduced, disability scores and quality-of-life changes, but they rarely ask patients to identify which specific activities, roles or periods of normal functioning have been restored. Consequently, the final transition from reduced disease frequency to restored meaningful time remains incompletely measured.

### 4.2. Clinical Meaning of Days Restored

The distinction between days with migraine and days meaningfully affected by migraine has an important implication for the interpretation of preventive treatment: a reduction in migraine days should not automatically be equated with an equivalent number of fully restored days. The clinical meaning of treatment response depends not only on how many migraine days are prevented, but also on whether the time gained is accompanied by recovery of normal physical, cognitive, occupational and social functioning.

The evidence reviewed in the present study supports this distinction. Premonitory and postdromal symptoms demonstrate that the clinically relevant consequences of an attack may extend beyond the headache phase [3,4,5]. Interictal anxiety and avoidance further demonstrate that migraine-related impairment may persist during periods without active headache [6]. Patient-reported measures such as MIDAS, MSQ and MIBS-4 capture additional dimensions of this burden that are not contained within the monthly migraine-day count [7,16,18].

This has implications for interpreting treatment response at the individual-patient level. Consider two patients who each experience a reduction of seven migraine days per month. The frequency endpoint assigns the same numerical response to both patients. However, if one patient resumes work, household activities, exercise and social commitments on most of the seven additional days, while the other continues to experience residual symptoms or anticipatory avoidance, the functional meaning of the treatment response is clearly different. The frequency endpoint alone cannot distinguish these scenarios.

The same consideration applies to patients with high-frequency or chronic migraine. In these populations, treatment may produce a substantial reduction in the number of migraine days without eliminating all disease-related consequences. The transition from chronic to episodic migraine may represent a clinically meaningful improvement in disease status, but it does not demonstrate that every day outside the remaining migraine days is fully restored. Functional recovery therefore requires direct assessment rather than inference from diagnostic category alone.

This distinction also has implications for communication of treatment benefit. Statements such as “seven fewer migraine days per month” are precise and clinically useful, but they do not describe the entire patient experience. A complementary description of functional recovery could potentially provide a more intuitive representation of benefit, particularly for patients for whom the principal treatment goal is to regain predictable participation in work, family, social and personal activities.

However, the concept of a restored day requires careful operational definition. A day should not be considered restored solely because it does not meet criteria for a migraine day. Similarly, restoration should not necessarily require complete absence of every symptom. The threshold for meaningful recovery may depend on the patient’s ability to perform predefined activities at an acceptable level without substantial migraine-related restriction. These criteria would need to be established empirically through patient-centred research.

Accordingly, “days restored” should currently be regarded as a clinically meaningful concept rather than a validated numerical endpoint. Its potential value is to shift the interpretation of preventive treatment from the reduction of disease frequency toward the recovery of meaningful time and function. This perspective complements rather than replaces established measures such as MMD, MIDAS, MSQ and MIBS-4 and provides a basis for developing future patient-centred outcome measures in migraine.

### 4.3. Clinical and Methodological Implications

The findings of this review have implications for both clinical interpretation and the design of future studies evaluating preventive treatment in migraine. The evidence does not indicate that conventional frequency-based outcomes should be abandoned. Rather, it suggests that their interpretation should be complemented by measures that capture the functional consequences of migraine and the extent to which these consequences are reduced with treatment.

The reviewed evidence suggests that patient-reported outcomes should be interpreted as complementary dimensions of treatment benefit rather than as interchangeable proxies for migraine frequency. Their classification as secondary outcomes in current trial guidance does not imply that they are clinically unimportant; rather, the key methodological issue is whether their results are consistently reported, interpreted alongside the primary frequency outcome, and incorporated into the overall assessment of patient-relevant benefit. Measures such as MIDAS, MSQ and MIBS-4 provide information about domains that are clinically distinct from migraine frequency [7,16,18]. Their inclusion allows treatment effects to be characterised more comprehensively and may help distinguish reduction in disease occurrence from improvement in the consequences of disease.

From a methodological perspective, future preventive-treatment studies could benefit from prospectively defining a core set of complementary outcomes. Such a framework could include migraine-day frequency, attack characteristics, acute medication use, disability, migraine-specific quality of life, interictal burden and functional productivity. The objective would not be to create an unnecessarily complex endpoint battery, but to ensure that the principal dimensions of treatment benefit are represented.

Taken together, the evidence supports a pragmatic methodological position: migraine-day frequency should remain a central outcome, but it should be interpreted within a broader framework of disability, quality of life, interictal burden and functional recovery. The proposed concept of migraine-affected or restored days represents a hypothesis-generating framework that may help guide this development, but prospective validation is required before it can be recommended as a clinical or regulatory endpoint.

### 4.4. Relationship to Current Clinical Trial Guidelines

An argument about the adequacy of trial endpoints must engage with the body that specifies them. The International Headache Society Clinical Trials Standing Committee publishes guidelines for controlled trials of preventive treatment in chronic migraine [31] and in episodic migraine [8], and these documents define the endpoints used across the programmes reviewed here.

The current International Headache Society guidelines therefore recognise a multidimensional approach to preventive-treatment outcomes. Both recommend changes in monthly migraine days or monthly migraine headache days as the primary efficacy endpoint, while also identifying responder rates, acute medication use, headache-related disability and health-related quality of life as important secondary outcomes [8,31]. The chronic migraine guideline additionally addresses medication overuse and assessment of comorbid depression and anxiety [31]. The central issue identified in the present review is therefore not that contemporary guidelines or trials recognise frequency alone, but that multidimensional outcomes may differ in how consistently they are measured, defined, reported and interpreted, and are not necessarily integrated into a common assessment of overall treatment benefit. A systematic review of preventive migraine trials similarly found substantial heterogeneity in the outcomes assessed and in endpoint definitions, despite the recurring use of multiple clinical and patient-reported domains [40].

Long-term evidence from the REGAIN programme also provides an important complementary perspective on preventive treatment outcomes. In the open-label extension of the REGAIN study, Pozo-Rosich et al. evaluated the long-term treatment experience with galcanezumab in patients with chronic migraine, providing evidence on outcomes during extended treatment beyond the controlled phase of the original trial [41]. Such long-term extension data complement short-term randomised trial evidence by addressing the durability of treatment effects and the clinical experience of continued preventive therapy.

Two domains identified in this review are not currently covered. The first is interictal burden. No instrument for assessing burden between attacks appears among the recommended secondary endpoints, and the MIBS-4 is not named in either guideline [8,31]. Given the evidence that interictal burden correlates only weakly with monthly migraine headache days, this is the clearest candidate for addition at the next revision. The second is peri-ictal impairment: neither guideline specifies how premonitory or postdromal symptoms should be recorded, so the recovery burden component of the framework proposed here has no standard method of measurement. These observations are summarised in Table 8.

The recent joint guidelines of the International Headache Society and the Italian Society for the Study of Headache reach a compatible conclusion from a different direction, noting that the evidence base is limited by a scarcity of head-to-head comparisons and of long-term outcome studies [42]. The proposal advanced in this review should therefore be read as a contribution to the next guideline revision rather than as a criticism of an absent standard.

### 4.5. A Candidate Operationalisation of Migraine-Affected Days

A conceptual framework that cannot be specified cannot be tested. We therefore set out one candidate operationalisation, not because we regard it as correct, but because a proposal that can be criticised is more useful than a term that can only be doubted.

Under this candidate operationalisation, a calendar day could be provisionally classified as migraine-affected if it satisfied any one of three conditions. First, the day meets existing criteria for a migraine day or a headache day as defined in the relevant trial protocol. Second, the day is contiguous with such a day, and the patient records premonitory or postdromal symptoms together with a reduction in usual activity meeting a prespecified threshold. Third, the day contains neither headache nor peri-ictal symptoms, but the patient records migraine-attributable restriction of planned activity, avoidance or anticipatory limitation that meets the same prespecified functional-impairment threshold.

Three rules would govern the candidate classification. A day satisfying more than one condition would be counted once, so that peri-ictal and interictal components would not be added to the same day. For headache-free days, attribution to migraine would require an explicit patient-reported indication that migraine was a principal cause of the reported restriction, avoidance or anticipatory limitation.

Discriminant validity should be assessed against constructs that may independently contribute to functional impairment but are not specific to migraine, particularly depression, anxiety, sleep disturbance and other chronic pain burden. Multivariable analyses should determine whether the proposed construct retains an independent association with patient-reported functional outcomes after adjustment for these potential confounders.

A headache-free day would not be classified as migraine-affected when another condition or circumstance provides a more plausible primary explanation for the reported functional restriction. Sensitivity analyses should further examine whether the classification remains stable when days with potentially competing causes are included or excluded.

The functional-impairment threshold should not be fixed arbitrarily at this conceptual stage. Instead, the same prespecified response format and recall period should be used for all participants, while the numerical or ordinal threshold should be established empirically during prospective validation. Candidate thresholds should be calibrated against an external anchor of patient-defined meaningful functional change and evaluated for reproducibility, discrimination and stability across relevant patient subgroups.

If subsequently established through prospective validation, the threshold should be prespecified and applied consistently across participants rather than determined separately for individual patients. If empirically validated, the resulting quantity could be examined alongside monthly migraine days rather than considered as a replacement for them, and its complement, the count of days on which no condition is met, gives the days restored measure discussed in Section 4.2.

Prospective validation should proceed in several stages. Test–retest reliability should first be examined during periods of relative clinical stability to determine whether the daily classification and aggregated construct are reproducible when migraine status has not materially changed. Responsiveness should then be evaluated longitudinally during preventive treatment, with change in the proposed construct compared with changes in established measures of migraine frequency and patient-reported benefit. Convergent validity should be examined against established measures of disability, migraine-related quality of life and interictal burden, whereas discriminant validity should be assessed against constructs that are related to functional impairment but not specifically attributable to migraine, including depression, anxiety, sleep disturbance and other chronic pain burden. Incremental validity should be tested by determining whether the proposed construct explains patient-reported functional or treatment-related outcomes beyond monthly migraine days (MMD) and monthly headache days (MHD). Finally, a minimal clinically important difference should be established using both anchor-based and distribution-based approaches, with the threshold linked to a patient-defined meaningful improvement in functioning.

Until these properties have been demonstrated prospectively, the proposed construct should not be used as an established trial endpoint. The operationalisation presented above should therefore be interpreted as a testable specification for future validation studies rather than as a validated measurement definition or empirical result.

### 4.6. Strengths and Limitations of the Review

This review has several strengths. First, it addresses migraine burden as a multidimensional construct rather than restricting the analysis to attack frequency. The framework integrates evidence concerning the temporal characteristics of migraine attacks, including premonitory and postdromal symptoms, interictal burden, disability, occupational and social functioning, quality of life and treatment-related outcomes. This approach allows findings that are usually reported in separate domains to be considered within a common clinical framework.

A second strength is the integration of evidence from different study designs. Population-based studies provide information on the broader burden of migraine, diary-based studies characterise symptoms occurring around individual attacks, patient-reported outcome studies assess disability and interictal burden, while randomised clinical trials and real-world cohorts provide complementary evidence regarding treatment-related changes. The inclusion of both controlled and observational evidence allows the distinction between treatment efficacy and treatment effectiveness to be maintained.

The review also has important limitations. It is a structured narrative review rather than a systematic review or meta-analysis. No formal risk-of-bias assessment was performed, no review protocol was registered, and study selection reflects the authors’ judgement regarding relevance to the framework developed in this article. Consequently, the review should be interpreted as a structured synthesis and conceptual analysis rather than as an exhaustive quantitative estimate of the evidence base. Publication bias within the preventive migraine literature was not formally assessed or quantified.

A second limitation concerns the heterogeneity of the evidence base. The studies considered differ in population characteristics, geographic setting, definitions of migraine frequency, follow-up duration, outcome measures and analytical approaches. This geographic heterogeneity is particularly relevant for the interpretation of interictal burden. The Eurolight findings provide important population-based evidence from Europe but should not be regarded as globally representative. More recent large studies from Japan, together with evidence from the United States, the European Union and Spain identified in a recent systematic review, provide broader geographic coverage; however, differences in instruments, sampling strategies and definitions of interictal burden limit direct quantitative comparison across settings.

A related limitation concerns the representativeness of some quantitative estimates presented in Table 5. Several measures of disability, productivity and health-related quality of life were derived from a tertiary headache-centre cohort and therefore may not be directly generalisable to community-based populations or to healthcare settings with different referral patterns. These data are presented as illustrative examples of the multidimensional functional burden of migraine rather than as population-level estimates.

This limitation is partly mitigated by the inclusion in the narrative synthesis of evidence from population-based studies conducted in other geographic and healthcare settings, including analyses from the United States and Germany, although differences in sampling strategies and outcome definitions preclude direct quantitative comparison across studies.

The interpretation of several findings also requires caution because some outcomes were derived from post hoc analyses. The interictal burden analyses and other secondary analyses were not necessarily part of the original hierarchical testing strategy and, where applicable, were not controlled for multiplicity. These findings should therefore be regarded as supportive and hypothesis-generating rather than as definitive evidence for causal relationships between individual outcome domains.

Potential sponsorship bias represents another consideration. A substantial proportion of the galcanezumab outcome literature discussed in this review, including pivotal trials and several analyses addressing disability, pain, functional outcomes and interictal burden [43,44,45], was sponsored by the manufacturer, and several publications included authors employed by the sponsor. This does not invalidate the findings, particularly because the studies were published in peer-reviewed journals with declared interests, but it represents a potential source of bias that should be considered when interpreting treatment-related evidence, especially for outcomes beyond the prespecified primary frequency endpoints.

The evidentiary hierarchy described above is therefore important when interpreting the sponsor-supported literature: prespecified primary randomised outcomes should not be treated as equivalent to secondary, exploratory or post hoc analyses, and neither should these trial-derived findings be considered interchangeable with observational real-world evidence. Independent replication of disability, functional and interictal findings would strengthen the evidence base. The investigator-initiated real-world cohorts included in the review provide complementary effectiveness evidence, although their observational design does not eliminate the methodological limitations inherent to non-randomised studies.

The use of galcanezumab as the principal treatment example also limits generalisability. This approach provides consistency because evidence from different outcome domains can be examined within the same treatment programme, but the findings cannot automatically be extrapolated to all CGRP-targeted monoclonal antibodies or to conventional oral preventive therapies [46,47]. The present review does not provide comparative efficacy estimates between individual CGRP-targeted agents or between these therapies and other preventive classes.

The real-world evidence has additional limitations related to both causal inference and population representativeness. The observational cohorts considered were predominantly conducted within European healthcare systems in which reimbursement and treatment-access criteria favour patients with relatively high attack frequency and multiple previous preventive-treatment failures. Because these studies generally lack randomised comparators and are susceptible to confounding, selection effects, regression to the mean and time-dependent changes, their findings cannot provide the same level of causal inference as randomised controlled trials. Nevertheless, they provide important complementary information on treatment persistence, tolerability, acute medication use and functional outcomes in routine practice. Their findings should therefore be interpreted primarily as evidence of effectiveness and treatment experience rather than as unbiased estimates of causal treatment efficacy. In addition, their populations should not be assumed to represent the wider population of people living with migraine. Similarly, much of the burden literature originates from high-income healthcare settings, which may influence both the availability of diagnosis and the extent to which migraine-related burden is systematically recorded.

Finally, and most importantly for the central proposition of this review, the proposed “migraine-affected days” construct has not yet been operationalised or validated. It is presented as a proposed conceptual and hypothesis-generating framework and research agenda rather than as a clinical or psychometric measurement instrument. The apparent novelty of the terminology should itself encourage caution: a new label should not be allowed to imply a level of empirical validation that the current evidence does not provide.

## 5. Conclusions

Migraine burden extends substantially beyond the number of monthly migraine or headache days recorded in clinical diaries. The evidence reviewed in this study demonstrates that migraine is a multidimensional and temporally extended condition in which burden may arise during the attack itself, during premonitory and postdromal periods, and between attacks through anticipatory anxiety, avoidance, impaired planning and restrictions in everyday life.

Monthly migraine days remain an important and clinically useful measure of preventive treatment response. However, they represent only one dimension of treatment benefit. Disability, quality of life, work and household productivity, acute medication use and interictal burden provide additional information that cannot be reliably inferred from migraine-day frequency alone.

The evidence from the galcanezumab programme illustrates the value of complementary outcome domains, but the underlying evidence should be interpreted according to study design. Randomised controlled trials provide the principal evidence that treatment can causally reduce migraine or headache frequency and can also demonstrate changes in prespecified patient-reported outcomes such as disability and migraine-specific quality of life. Prospective real-world cohorts provide complementary evidence regarding treatment effectiveness in routine practice, including treatment persistence, acute medication use, disability and changes in migraine chronicity. Because these observational studies are not randomised, their findings cannot provide the same level of causal inference as controlled trials and should not be interpreted as equivalent estimates of treatment efficacy.

The central implication of this review is therefore not that migraine-day frequency should be replaced, but that it should be interpreted within a broader framework of patient-relevant recovery. The distinction between “days reduced” and “days restored” may be particularly useful in this context. A day without a qualifying migraine attack is not necessarily equivalent to a day of fully restored function, and the number of migraine days prevented does not directly quantify the amount of meaningful time regained by the patient.

The proposed “migraine-affected days” construct is proposed as a conceptual framework for addressing the gap between conventional frequency-based measurement and patients’ lived experience. It integrates attack burden, recovery burden and functional burden while preserving established migraine-day and headache-day outcomes. At present, however, the construct has not been operationalised or validated and should therefore be regarded strictly as a hypothesis-generating framework and research agenda, rather than as a clinical, psychometric or regulatory endpoint.

Future prospective studies should determine whether the additional domains identified in this review can be measured reliably, whether they provide information beyond established endpoints, and whether treatment-related changes in these domains correspond to patient-defined meaningful recovery. Randomised studies will be particularly important for determining the causal relationship between preventive treatment and changes in these outcomes, whereas longitudinal real-world cohorts will remain essential for characterising persistence, tolerability, medication use, functional outcomes and treatment experience under routine clinical conditions.

Ultimately, the clinically relevant question is not only how many migraine days treatment prevents, but what happens to the time that migraine no longer occupies. Moving from the measurement of days reduced toward the measurement of meaningful days restored may provide a more patient-centred understanding of preventive treatment benefit, provided that such a framework is subjected to rigorous prospective validation.

## Figures and Tables

**Table 1:** Selected global epidemiological metrics for migraine and headache disorders from the Global Burden of Disease Study 2021.

Epidemiological Metric	Finding	Source
Estimated global number of people living with migraine, 2021	1.2 billion (95% UI, 1.0–1.3 billion)	[1]
Global age-standardised prevalence rate of migraine, 2021	14,246.6 per 100,000 (95% UI, 12,194.1–16,378.7)	[1]
Global number of migraine YLDs, 2021	43.4 million (95% UI, 6.7–95.1 million)	[1]
Global age-standardised YLD rate for migraine, 2021	532.7 per 100,000 (95% UI, 80.6–1167.7)	[1]
Change in global migraine prevalence, 1990–2021	+58.1%	[1]
Change in migraine YLDs, 1990–2021	+58.2%	[1]
Global prevalence rank of headache disorders	2nd most prevalent disease globally	[1]
Estimated number of people with headache disorders, 2021	2.8 billion (95% UI, 2.6–3.0 billion)	[1]
Global YLD burden of headache disorders, 2021	48.0 million (95% UI, 9.8–101.0 million)	[1]
Global YLD rank of headache disorders	3rd leading cause of YLDs	[1]
Global prevalence rank of migraine	7th among specific conditions	[1]
Age/sex group with highest migraine burden	Women aged 30–44 years	[1]
Global migraine prevalence, 1990	732.6 million (95% UI, 624.6–847.1 million)	[1]
Global age-standardised prevalence rate, 1990	14,027.7 per 100,000	[1]
Global migraine YLDs, 1990	27.4 million (95% UI, 4.1–60.3 million)	[1]

**Table 2:** Conventional frequency-based measures and additional dimensions of migraine burden identified in the reviewed evidence.

Dimension or Characteristic	Evidence-Based Measure or Characteristic	Main Information Captured	Limitation of Frequency-Only Assessment
Migraine frequency	Monthly migraine days (MMD); monthly headache days (MHD)	Frequency of migraine/headache occurrence	Does not quantify the severity or duration of individual attacks or burden outside headache periods
Premonitory phase	Premonitory/prodromal symptoms, including fatigue, mood or activity changes, difficulty concentrating, neck stiffness, food cravings, yawning and increased sensory sensitivity [3,4]	Symptoms and functional changes preceding headache onset	May occur before a formally recorded migraine day and therefore may not be captured by frequency-based measures
Headache phase	Pain intensity and headache duration; 4-point categorical headache severity scale, 11-point numerical rating scale (NRS; 0 = no pain, 10 = worst possible pain), or 100 mm visual analogue scale (VAS) [2,8]	Headache severity/peak pain intensity and duration of the active headache phase	Equal numbers of migraine days may represent different total pain burdens [2]
Postdrome	Non-headache symptoms following headache resolution, including fatigue, cognitive difficulties, neck symptoms and residual head discomfort [5]	Recovery-related symptoms and delayed return to usual functioning	Symptoms may persist after headache resolution and may therefore extend beyond the recorded headache day [5]
Interictal burden	Migraine Interictal Burden Scale (MIBS-4); interictal symptoms, anxiety, avoidance and lifestyle compromise [6,7]	Burden occurring between migraine attacks	Shows only low correlation with monthly migraine headache days, indicating that interictal burden is not captured adequately by frequency alone [6,7]
Overall disease burden	Combination of frequency, attack characteristics, recovery-related symptoms and functional/interictal consequences	Broader temporal and functional impact of migraine	Cannot be represented by a single frequency measure alone

**Table 3:** Clinical characteristics of the premonitory and prodromal and postdromal phases of migraine identified in the reviewed evidence.

Migraine Phase	Characteristic Findings	Quantitative Evidence	Reference
Premonitory phase	Fatigue, mood changes, difficulty concentrating, neck stiffness, nausea, sensory sensitivity, yawning and food craving	Population-based studies: pooled prevalence of at least one premonitory symptom 29% (95% CI 8–63%); clinic-based studies: pooled relative frequency 66% (95% CI 45–82%). Estimates showed substantial heterogeneity.	[9]
Premonitory phase	Fatigue, neck stiffness, mood changes, concentration difficulties and other non-headache symptoms	In clinic-based studies included in the meta-analysis, pooled relative frequencies were 49% for fatigue, 46% for neck stiffness, 37% for mood change, and 30% for concentration difficulties; pooled estimates were also available for nausea (29%), photophobia (29%), phonophobia (26%), yawning (22%), depressive symptoms (19%), irritability (16%) and food craving (11%).	[9]
Premonitory phase	Fatigue, neck pain, nausea, concentration difficulties and sensory symptoms	In the REFORM study, 448/632 participants (71%) reported premonitory symptoms when prompted and unprompted assessment methods were considered together. With prompting, fatigue was reported by 39.9%, difficulty concentrating by 35.0%, and neck pain by 33.2%.	[10]
Premonitory phase	Fatigue/weariness, difficulty concentrating and stiff neck	In the electronic-diary study by Giffin et al., the most common premonitory symptoms were fatigue/weariness in 72%, difficulty concentrating in 51%, and stiff neck in 50% of attacks with warning features.	[4]
Premonitory phase	Photophobia, fatigue, neck pain, phonophobia, difficulty thinking/concentrating and dizziness	In the PRODROME screening cohort, 920 participants recorded 4802 qualifying premonitory events. Photophobia occurred in 57.2%, fatigue in 50.1%, neck pain in 41.9%, phonophobia in 33.9%, difficulty thinking/concentrating in 30.0%, and dizziness in 27.8% of qualifying events. 81.5% of qualifying events were followed by headache within 1–6 h.	[11]
Postdromal phase	Fatigue, concentration difficulties, mood changes, yawning and residual symptoms after headache resolution	A systematic review and meta-analysis reported postdromal symptoms in a mean of 86% (95% CI 71–94%) of participants in four clinic-based studies. The only population-based study reported 97%, although the definition of postdrome was broad.	[12]
Postdromal phase	Fatigue, concentration difficulties and mood changes	In the meta-analysis of individual postdromal symptoms, fatigue occurred in 52% (95% CI 44–60%), concentration difficulties in 35% (95% CI 14–65%), and mood changes in 29% (95% CI 9–64%). Estimates were characterised by substantial heterogeneity.	[12]
Postdromal phase	Fatigue/weariness, difficulty concentrating, stiff neck and residual head discomfort	In the 3-month electronic-diary study by Giffin et al., 97/120 participants (81%) reported at least one non-headache postdromal symptom. Fatigue/weariness occurred in 88%, difficulty concentrating in 56%, and stiff neck in 42% of postdromes.	[5]
Postdromal phase	Functional recovery after headache resolution	In the same prospective study, 93% of attacks returned to normal functioning within 24 h after spontaneous resolution of headache; however, health-state scores remained lower during the postdrome than during the interictal period.	[5]

**Table 4:** Quantitative evidence describing interictal burden in migraine across different populations and study designs.

Evidence Source	Population/Setting	Quantitative Findings	Interpretation
Eurolight project	6455 participants with headache from 9 European countries; 2959 classified as having migraine	Interictal symptoms were reported by 26.0% of participants with migraine; interictal anxiety by 10.6%; avoidance/lifestyle compromise by 14.8%. Mean time spent in the interictal state was 317 days/year.	Large population-based European evidence demonstrating that migraine-related burden may persist between headache attacks.
Eurolight project	European population-based sample	Compared with tension-type headache, interictal anxiety was associated with OR 3.8 (95% CI, 3.0–4.8) and interictal avoidance with OR 3.5 (95% CI, 2.9–4.3) among participants with migraine.	Demonstrates that selected interictal consequences may be more frequent in migraine than in tension-type headache, but the estimates are European.
OVERCOME (Japan)	17,071 respondents with migraine in a population-based national web survey	41.5% reported moderate-to-severe interictal burden according to MIBS-4. Between headache days, 26.8%reported worrying about planning social or leisure activities at least some of the time; 19.0% reported that headaches affected work or school at least some of the time when they did not have a headache.	Large non-European population-based evidence supporting the presence of clinically relevant interictal burden.
OVERCOME (Japan), second study	19,590 adults with migraine surveyed in 2023	47.1% had MIBS-4 scores ≥3, corresponding to moderate-to-severe interictal burden. Participants with higher MIBS-4 scores had greater disability, lower migraine-specific quality of life and greater work/activity impairment, including within strata with lower ictal burden.	Contemporary Japanese evidence indicates that interictal burden is not confined to patients with high ictal burden.
Phase 3 preventive-treatment cohort	462 participants with episodic or chronic migraine	MIBS-4 showed Spearman r = 0.21 with monthly migraine headache days at baseline and r = 0.32 at month 6.	Interictal burden is related to, but not fully captured by, migraine-day frequency.
Systematic review of interictal-burden instruments	29 studiesacross multiple populations and settings	MIBS-4 was used in 26 studies, the Eurolight questionnaire in 1 study, and the Fear of Attacks Inventory in 2 studies. Large cohorts included populations from Japan, the United States, the European Union and Spain.	The contemporary evidence base is geographically broader than Eurolight alone, although measurement properties remain incompletely characterised across instruments and settings.

**Table 5:** Selected evidence describing the functional, occupational and social burden of migraine.

Domain	Finding	Reference
Missed work/school days—chronic migraine	57.2 ± 87.6 days/year	[19]
Missed work/school days—episodic migraine	6.8 ± 18.0 days/year	[19]
Days with ≥50% productivity reduction—chronic migraine	82.0 ± 97.6 days/year	[19]
Days with ≥50% productivity reduction—episodic migraine	13.6 ± 31.2 days/year	[19]
MIDAS score—chronic migraine	98.4 ± 72.3	[19]
MIDAS score—episodic migraine	15.5 ± 17.7	[19]
HIT-6 score—chronic migraine	66.1 ± 8.4	[19]
HIT-6 score—episodic migraine	58.7 ± 10.1	[19]
SF-36 general health—chronic migraine	39.9 ± 14.7	[19]
SF-36 general health—episodic migraine	66.2 ± 18.2	[19]
Mean annual cost per person attributable to migraine in Europe	€1222; 93% indirect costs	[20]
Estimated annual European cost attributable to migraine	€111 billion	[20]
Proportion of total headache economic burden attributable to migraine	64%	[20]

**Table 6:** Complementary outcome measures required to characterise the multidimensional burden of migraine.

Outcome Domain	Representative Measure	Information Provided	Relationship To Migraine-Day Frequency
Attack frequency	MMD; MHD	Frequency of migraine/headache occurrence	Directly quantified
Disability	MIDAS	Lost or reduced productivity in work/school, household and social/leisure activities	Complementary to frequency [16]
Headache impact	HIT-6	Patient-reported overall impact of headache	Complementary
Migraine-specific quality of life	MSQ v2.1	Role-function restrictive, role-function preventive and emotional-function domains	Complementary [18]
Interictal burden	MIBS-4	Burden occurring between migraine attacks	Only low correlation with MHD [7]
Attack severity and duration	Diary-based severity and duration measures	Intensity and temporal extent of individual attacks	Not represented by frequency alone
Acute medication use	Acute medication days	Frequency of reliance on acute treatment	Related but distinct treatment-burden domain
Work productivity	WPAI	Absenteeism and presenteeism	Not captured by MMD/MHD alone

**Table 7:** Complementary domains for evaluating preventive treatment response in migraine.

Outcome Domain	Representative Endpoint	Aspect of Treatment Response Assessed
Migraine frequency	MMD/MHD	Reduction in the number of migraine or headache days
Disability	MIDAS	Reduction in days with missed or substantially reduced productivity
Headache impact	HIT-6	Change in overall patient-perceived headache impact
Migraine-specific quality of life	MSQ v2.1	Improvement in role and emotional functioning
Interictal burden	MIBS-4	Reduction in burden occurring between attacks
Attack characteristics	Attack duration/severity	Reduction in the temporal and symptomatic burden of individual attacks
Acute treatment use	Acute medication days	Reduction in reliance on acute symptomatic treatment
Work productivity	WPAI	Changes in absenteeism and presenteeism
Overall clinical benefit	Combined assessment	Concordance between frequency reduction and improvement in functional and patient-reported outcomes

**Table 8:** Coverage of migraine burden domains in current preventive-treatment trial guidelines and the proposed framework.

Migraine Burden Domain	Current Guideline Coverage	Representative Outcome/Measure	Limitation Identified in the Present Review
Migraine frequency	Recommended as a core efficacy outcome	Monthly migraine days (MMD)/monthly migraine headache days (MHD)	Captures frequency but not the full temporal and functional burden of individual attacks
Responder status	Recommended	Proportion of participants achieving predefined reductions in migraine days	Describes frequency response but does not establish the extent of functional recovery
Acute medication use	Recommended	Acute medication days/medication use	Captures treatment-related medication burden but not broader interictal or peri-ictal consequences
Headache-related disability	Recommended as a secondary outcome	MIDAS and related disability measures	Captures functional consequences but does not quantify premonitory or postdromal impairment across individual days
Health-related quality of life	Recommended as a secondary outcome	Migraine-specific quality-of-life measures	Provides a broader patient-reported perspective but is not expressed as the number of days affected or restored
Medication overuse	Specifically addressed in chronic migraine guidance	Medication-overuse assessment	Identifies an important comorbidity/burden domain but does not quantify interictal or peri-ictal functional burden
Depression and anxiety	Addressed as comorbid conditions, particularly in chronic migraine guidance	Standard depression/anxiety assessment	Important for interpretation of burden but does not directly measure migraine-attributable interictal restriction
Interictal burden	Not specifically included as a recommended secondary endpoint	MIBS-4	May remain clinically relevant during headache-free periods and shows only weak correlation with monthly migraine headache days
Premonitory and postdromal impairment	No standard method specified	Patient-reported premonitory/postdromal symptoms and recovery	Current guidelines do not specify how peri-ictal impairment should be incorporated into treatment-response assessment
Migraine-affected days/days restored	Not established as a guideline endpoint	Proposed conceptual and hypothesis-generating framework	Requires operational definition and prospective validation before clinical or regulatory use

## Data Availability

No new data were created or analysed in this study. Data sharing is not applicable to this article.

## References

[1] Wijeratne T., Oh J., Kim S., Yim Y., Kim M.S., Shin J.I., Oh Y.-S., Jung R., Smith L., GBD 2021 Headache Collaborators (2025). Global, regional, and national burden of headache disorders, 1990–2021, with forecasts to 2050: A Global Burden of Disease study 2021. Cell Rep. Med..

[2] Ailani J., Andrews J.S., Rettiganti M., Nicholson R.A. (2020). Impact of galcanezumab on total pain burden: Findings from phase 3 randomized, double-blind, placebo-controlled studies in patients with episodic or chronic migraine (EVOLVE-1, EVOLVE-2, and REGAIN trials). J. Headache Pain.

[3] Headache Classification Committee of the International Headache Society (IHS) (2018). The International Classification of Headache Disorders, 3rd edition. Cephalalgia.

[4] Giffin N.J., Ruggiero L., Lipton R.B., Silberstein S.D., Tvedskov J.F., Olesen J., Altman J., Goadsby P.J., Macrae A. (2003). Premonitory symptoms in migraine: An electronic diary study. Neurology.

[5] Giffin N.J., Lipton R.B., Silberstein S.D., Olesen J., Goadsby P.J. (2016). The migraine postdrome: An electronic diary study. Neurology.

[6] Lampl C., Thomas H., Stovner L.J., Tassorelli C., Katsarava Z., Láinez J.M., Lantéri-Minet M., Rastenyte D., Ruiz de la Torre E., Andrée C. (2016). Interictal burden attributable to episodic headache: Findings from the Eurolight project. J. Headache Pain.

[7] Lampl C., Lipton R.B., Lipsius S., Ford J.H., Dell’Agnello G., Viktrup L., Buse D.C. (2026). Evaluating the relationship between the 4-item migraine interictal burden scale and other patient-reported outcome measures: Analysis from a phase 3 migraine prevention study. Front. Neurol..

[8] Diener H.C., Tassorelli C., Dodick D.W., Silberstein S.D., Lipton R.B., Ashina M., Becker W.J., Ferrari M.D., Goadsby P.J., Pozo-Rosich P. (2020). Guidelines of the International Headache Society for Controlled Trials of Preventive Treatment of Migraine Attacks in Episodic Migraine in Adults. Cephalalgia.

[9] Eigenbrodt A.K., Christensen R.H., Ashina H., Iljazi A., Christensen C.E., Steiner T.J., Lipton R.B., Ashina M. (2022). Premonitory symptoms in migraine: A systematic review and meta-analysis of observational studies reporting prevalence or relative frequency. J. Headache Pain.

[10] Thuraiaiyah J., Ashina H., Christensen R.H., Al-Khazali H.M., Wiggers A., Amin F.M., Steiner T.J., Ashina M. (2024). Premonitory symptoms in migraine: A REFORM Study. Cephalalgia.

[11] Schwedt T.J., Lipton R.B., Goadsby P.J., Chiang C.C., Klein B.C., Hussar C., Liu C., Yu S.Y., Finnegan M., Trugman J.M. (2025). Characterizing Prodrome (Premonitory Phase) in Migraine: Results From the PRODROME Trial Screening Period. Neurol. Clin. Pract..

[12] Christensen R.H., Eigenbrodt A.K., Ashina H., Steiner T.J., Ashina M. (2023). What proportion of people with migraine report postdromal symptoms? A systematic review and meta-analysis of observational studies. Cephalalgia.

[13] Awaki E., Takeshima T., Matsumori Y., Hirata K., Miyazaki N., Takemura R., Osaga S., Tanizawa Y., Komori M. (2024). Impact of Migraine on Daily Life: Results of the Observational survey of the Epidemiology, Treatment, and Care of Migraine (OVERCOME [Japan]) Study. Neurol. Ther..

[14] Takizawa T., Danno D., Ishii R., Suzuki S., Miura M., Tanizawa Y., Osaga S., Okada M., Hashimoto C., Komori M. (2025). The dual impact of ictal and interictal burden in migraine: An analysis from the ObserVational survey of the Epidemiology, tReatment, and Care Of MigrainE (OVERCOME) Japan second study. J. Headache Pain.

[15] Angerhöfer C., Fuse Y., Van Langenhove S., Canning T., von Alvensleben A.C., Ahmad S., Murillo A., Boada L., Roque M.M., MaassenVanDenBrink A. (2026). Interictal burden in migraine: A systematic review of conceptual domains and measurement properties of instruments. J. Headache Pain.

[16] Lipton R.B., Stewart W.F., Sawyer J., Edmeads J.G. (2001). Clinical utility of an instrument assessing migraine disability: The Migraine Disability Assessment (MIDAS) questionnaire. Headache.

[17] Kosinski M., Bayliss M.S., Bjorner J.B., Ware J.E., Garber W.H., Batenhorst A., Cady R., Dahlöf C.G.H., Dowson A., Tepper S. (2003). A six-item short-form survey for measuring headache impact: The HIT-6. Qual. Life Res..

[18] Cole J.C., Lin P., Rupnow M.F.T. (2007). Validation of the Migraine-Specific Quality of Life Questionnaire version 2.1 (MSQ v. 2.1) for patients undergoing prophylactic migraine treatment. Qual. Life Res..

[19] Berra E., Sances G., De Icco R., Avenali M., Berlangieri M., De Paoli I., Bolla M., Allena M., Ghiotto N., Guaschino E. (2015). Cost of Chronic and Episodic Migraine: A Pilot Study from a Tertiary Headache Centre in Northern Italy. J. Headache Pain.

[20] Linde M., Gustavsson A., Stovner L.J., Steiner T.J., Barré J., Katsarava Z., Láinez J.M., Lampl C., Lantéri-Minet M., Rastenyte D. (2012). The cost of headache disorders in Europe: The Eurolight project. Eur. J. Neurol..

[21] Thomas H., Kothari S.F., Husøy A., Jensen R.H., Katsarava Z., Tinelli M., Steiner T.J. (2021). The Relationship between Headache-Attributed Disability and Lost Productivity: 2. Empirical Evidence from Population-Based Studies in Nine Disparate Countries. J. Headache Pain.

[22] Buse D.C., Fanning K.M., Reed M.L., Murray S., Dumas P.K., Adams A.M., Lipton R.B. (2019). Life with migraine: Effects on relationships, career, and finances from the Chronic Migraine Epidemiology and Outcomes (CaMEO) study. Headache.

[23] Seddik A.H., Branner J.C., Ostwald D.A., Schramm S.H., Bierbaum M., Katsarava Z. (2020). The socioeconomic burden of migraine: An evaluation of productivity losses due to migraine headaches based on a population study in Germany. Cephalalgia.

[24] Seng E.K., Muenzel E.J., Shapiro R.E., Buse D.C., Reed M.L., Zagar A.J., Ashina S., Hutchinson S., Nicholson R.A., Lipton R.B. (2025). Development of the Migraine-Related Stigma (MiRS) questionnaire: Results of the OVERCOME (US) study. Headache.

[25] Dodick D.W., Loder E.W., Manack Adams A., Buse D.C., Fanning K.M., Reed M.L., Lipton R.B. (2016). Assessing barriers to chronic migraine consultation, diagnosis, and treatment: Results from the Chronic Migraine Epidemiology and Outcomes (CaMEO) study. Headache.

[26] Lipton R.B., Bigal M.E., Diamond M., Freitag F., Reed M.L., Stewart W.F., AMPP Advisory Group (2007). Migraine prevalence, disease burden, and the need for preventive therapy. Neurology.

[27] Buse D.C., Sakai F., Matharu M., Reed M.L., Fanning K., Dabruzzo B., Lipton R.B. (2024). Characterizing gaps in the preventive pharmacologic treatment of migraine: Multi-country results from the CaMEO-I study. Headache.

[28] Hutchinson S., Johnston E., Nicholson R.A., Reed M.L., Buse D.C., Zagar A., Kim G., Muenzel E.J., Pearlman E.M., Lipton R.B. (2026). Unmet treatment needs in migraine: Results of the OVERCOME (US) study. Headache.

[29] Hepp Z., Dodick D.W., Varon S.F., Chia J., Matthew N., Gillard P., Hansen R.N., Devine E.B. (2017). Persistence and switching patterns of oral migraine prophylactic medications among patients with chronic migraine: A retrospective claims analysis. Cephalalgia.

[30] Lipton R.B. (2009). Tracing transformation: Chronic migraine classification, progression, and epidemiology. Neurology.

[31] Tassorelli C., Diener H.-C., Dodick D.W., Silberstein S.D., Lipton R.B., Ashina M., Becker W.J., Ferrari M.D., Goadsby P.J., Pozo-Rosich P. (2018). Guidelines of the International Headache Society for controlled trials of preventive treatment of chronic migraine in adults. Cephalalgia.

[32] Stauffer V.L., Dodick D.W., Zhang Q., Carter J.N., Ailani J., Conley R.R. (2018). Evaluation of Galcanezumab for the Prevention of Episodic Migraine: The EVOLVE-1 Randomized Clinical Trial. JAMA Neurol..

[33] Skljarevski V., Matharu M., Millen B.A., Ossipov M.H., Kim B.-K., Yang J.Y. (2018). Efficacy and safety of galcanezumab for the prevention of episodic migraine: Results of the EVOLVE-2 phase 3 randomized controlled clinical trial. Cephalalgia.

[34] Detke H.C., Goadsby P.J., Wang S., Friedman D.I., Selzler K.J., Aurora S.K. (2018). Galcanezumab in chronic migraine: The randomized, double-blind, placebo-controlled REGAIN study. Neurology.

[35] Mulleners W.M., Kim B.-K., Láinez M.J.A., Lantéri-Minet M., Pozo-Rosich P., Wang S., Tockhorn-Heidenreich A., Detke H.C. (2020). Safety and efficacy of galcanezumab in patients for whom previous migraine preventive medication from two to four categories had failed (CONQUER): A multicentre, randomised, double-blind, placebo-controlled, phase 3b trial. Lancet Neurol..

[36] Ament M., Day K., Stauffer V.L., Skljarevski V., Rettiganti M., Pearlman E., Aurora S.K. (2021). Effect of galcanezumab on severity and symptoms of migraine in phase 3 trials in patients with episodic or chronic migraine. J. Headache Pain.

[37] Ford J.H., Ayer D.W., Zhang Q., Carter J.N., Leroux E., Skljarevski V., Aurora S.K., Tockhorn-Heidenreich A., Lipton R.B. (2019). Two randomized migraine studies of galcanezumab: Effects on patient functioning and disability. Neurology.

[38] Vernieri F., Altamura C., Brunelli N., Costa C.M., Aurilia C., Egeo G., Fofi L., Favoni V., Pierangeli G., Lovati C. (2021). Galcanezumab for the prevention of high frequency episodic and chronic migraine in real life in Italy: A multicenter prospective cohort study (the GARLIT study). J. Headache Pain.

[39] Vernieri F., Brunelli N., Marcosano M., Aurilia C., Egeo G., Lovati C., Favoni V., Perrotta A., Maestrini I., Rao R. (2023). Maintenance of response and predictive factors of 1-year GalcanezumAb treatment in real-life migraine patients in Italy: The multicenter prospective cohort GARLIT study. Eur. J. Neurol..

[40] Ferreira V.L., Mainka F.F., Wiens A., Pontarolo R. (2023). Effectiveness of calcitonin gene-related peptide monoclonal antibodies in the prevention of migraine: A systematic review and meta-analysis of observational cohort studies. Clin. Drug Investig..

[41] Pozo-Rosich P., Detke H.C., Wang S., Doležil D., Li L.Q., Aurora S.K., Reuter U. (2022). Long-term treatment with galcanezumab in patients with chronic migraine: Results from the open-label extension of the REGAIN study. Curr. Med. Res. Opin..

[42] Ornello R., Caponnetto V., Ahmed F., Al-Khazali H.M., Ambrosini A., Ashina S., Baraldi C., Bellotti A., Brighina F., Calabresi P. (2025). Evidence-based guidelines for the pharmacological treatment of migraine, summary version. Cephalalgia.

[43] Ford J., Tassorelli C., Leroux E., Wang S., Ayer D., Nichols R., Detke H. (2021). Changes in patient functioning and disability: Results from a phase 3, double-blind, randomized, placebo-controlled clinical trial evaluating galcanezumab for chronic migraine prevention (REGAIN). Qual. Life Res..

[44] Ford J.H., Kurth T., Starling A.J., Ayer D.W., Wietecha L.A., Port M.D., Rettiganti M., Ruff D.D. (2020). Migraine Headache Day Response Rates and the Implications to Patient Functioning: An Evaluation of 3 Randomized Phase 3 Clinical Trials of Galcanezumab in Patients With Migraine. Headache.

[45] Lipton R.B., Buse D.C., Sandoe C.H., Ford J.H., Hand A.L., Jedynak J.P., Port M.D., Detke H.C. (2023). Changes in migraine interictal burden following treatment with galcanezumab: Results from a phase III randomized, placebo-controlled study. Headache.

[46] Reuter U., Ehrlich M., Gendolla A., Heinze A., Klatt J., Wen S., Hours-Zesiger P., Nickisch J., Sieder C., Hentschke C. (2022). Erenumab versus topiramate for the prevention of migraine: A randomised, double-blind, active-controlled phase 4 trial. Cephalalgia.

[47] Lipton R.B. (2024). Preventive treatment of migraine. Continuum.

